# Individual Differences in Subjective Utility and Risk Preferences: The Influence of Hedonic Capacity and Trait Anxiety

**DOI:** 10.3389/fpsyt.2017.00088

**Published:** 2017-05-22

**Authors:** Jonathon R. Howlett, Martin P. Paulus

**Affiliations:** ^1^Department of Psychiatry, University of California San Diego, La Jolla, CA, USA; ^2^Laureate Institute for Brain Research, Tulsa, OK, USA

**Keywords:** depression, anxiety, decision-making, behavioral economics, reward, risk preferences, counterfactual thinking

## Abstract

Individual differences in decision-making are important in both normal populations and psychiatric conditions. Variability in decision-making could be mediated by different subjective utilities or by other processes. For example, while traditional economic accounts attribute risk aversion to a concave subjective utility curve, in practice other factors could affect risk behavior. This distinction may have important implications for understanding the biological basis of variability in decision-making and for developing interventions to improve decision-making. Another aspect of decision-making that may vary between individuals is the sensitivity of subjective utility to counterfactual outcomes (outcomes that could have occurred, but did not). We investigated decision-making in relation to hedonic capacity and trait anxiety, two traits that relate to psychiatric conditions but also vary in the general population. Subjects performed a decision-making task, in which they chose between low- and high-risk gambles to win 0, 20, or 40 points on each trial. Subjects then rated satisfaction after each outcome on a visual analog scale, indicating subjective utility. Hedonic capacity was positively associated with the subjective utility of winning 20 points but was not associated with the concavity of the subjective utility curve (constructed using the mean subjective utility of winning 0, 20, or 40 points). Consistent with economic theory, concavity of the subjective utility curve was associated with risk aversion. Hedonic capacity was independently associated with risk seeking (i.e., not mediated by the shape of the subjective utility curve), while trait anxiety was unrelated to risk preferences. Contrary to our expectations, counterfactual sensitivity was unrelated to hedonic capacity and trait anxiety. Nevertheless, trait anxiety was associated with a self-report measure of regret-proneness, suggesting that counterfactual influences may occur via a pathway that is separate from immediate counterfactual processing biases. Taken together, our results show that hedonic capacity but not trait anxiety affects risk-taking through a mechanism that appears independent of the shape of the subjective utility curve, while hedonic capacity and trait anxiety do not affect the influence of counterfactual outcomes on subjective utility. The results have implications for understanding the underlying mechanisms of variable decision-making and for developing interventions to improve decision-making.

## Introduction

Much of the traditional economic literature on decision-making concerns the behavior of a hypothetical perfectly rational agent, sometimes known as “Homo Economicus” (1). Such an agent would make decisions to maximize *subjective utility*, which is defined in terms of personal preferences regarding various possible decision outcomes. However, there has been increasing interest in deviations from rational decision-making in real humans (2) and in individual variability in decision-making (3).

One important area of individual differences in decision-making is propensity to take risks. Risk is defined economically as the mathematical variance of all possible outcomes of a decision (4). When choosing between two options with identical average expected outcome, many individuals prefer to choose the less risky option (i.e., these individuals are risk averse rather than risk seeking). In order to explain risk aversion, Daniel Bernoulli proposed that the subjective utility of an outcome is not directly proportional to the amount of money received, but instead that the subjective utility of an amount of money grows more slowly than the total amount of money (5). Equivalently, the subjective utility curve (with amount of reward, e.g., money, on the *x*-axis plotted against subjective utility on the *y*-axis) has a concave shape. Individual differences in risk aversion or risk seeking could therefore be explained in terms of different shapes of the subjective utility curve. However, it is also possible for risk behavior to vary independently from the shape of the subjective utility curve, which has been referred to as “pure” risk aversion or risk seeking (6).

Subjective utility can be influenced by factors other than objective reward magnitude. A large body of research has shown that the subjective utility of an outcome is also influenced by *counterfactual outcomes*, i.e., outcomes that could have occurred, but did not (7–10). When the actual outcome is better than the counterfactual outcome, the subjective utility of the actual outcome is enhanced; when the actual outcome is worse than the counterfactual outcome, the subjective utility of the actual outcome is lessened. When the actual outcome is worse than the counterfactual outcome as a result of an individual’s own actions, that individual tends to feel *regret*, which is a particular counterfactual-related emotion. There are significant individual differences in proneness to regret (11), suggesting that subjective utility may be differentially sensitive to counterfactual influences.

One area of particular interest is disordered decision-making in psychiatric conditions (12). At the same time, given the high prevalence of psychiatric conditions such as major depressive disorder [lifetime prevalence 16.6% (13)] and anxiety disorders [lifetime prevalence 28.8% (13)] and the dimensional nature of these disorders [with many more individuals exhibiting symptoms below the threshold for a clinical diagnosis (14)], there is likely to be a continuum between decision-making dysfunctions in psychiatric populations and normal variation in the general population. A better understanding of the sources of individual variation in decision-making could lead to better assessments and treatments of psychiatric conditions. The individual variability in decision-making discussed above (including different subjective utility curves, different risk behaviors, and different sensitivity of subjective utility to counterfactual outcomes) may be associated with traits related to clinical or subclinical psychiatric symptoms. For example, a large body of literature implicates deficient reward processing in depression (15), and this may manifest in an altered subjective utility function. Importantly, depression is a multidimensional construct and reward deficits may be linked more specifically to the symptom of anhedonia than to depression severity overall (16, 17). Clinically significant anhedonia in depression may exist on a continuum with low hedonic capacity (i.e., low capacity to take pleasure in typically rewarding experiences) in non-clinical populations (18). Hedonic capacity may affect risk behavior by influencing the shape of the subjective utility curve. Hedonic capacity may be related to reward sensitivity, which is considered to be a component of impulsivity (19) [which is itself linked to risk seeking behavior (3)]. While hedonic capacity may influence risk taking via its effect on the subjective utility curve, trait anxiety may be associated with risk aversion through a mechanism independent of the shape of the subjective utility curve. Anxious individuals are likely to exaggerate the probability of a negative outcome (3, 20) or focus on possible threats as opposed to opportunities (21). Anxious individuals are generally averse to uncertainty (of which risk is one form) (12). Anxiety may also affect decision-making via the manner in which an individual frames a situation (21) or by intensifying the influence of an externally provided frame (22). The effects of stress on decision-making may be complex and may depend on whether stress is acute or chronic; in some cases, acute stress may lead to increases in risky decisions (23) and may impair reward valuation (24).

In addition to direct influences on subjective utility and on risk behavior, hedonic capacity and trait anxiety may alter the extent to which subjective utility is sensitive to counterfactual influences. One study found a positive correlation between regret-proneness measured by a self-report scale (Regret Scale) and severity of depressive symptoms in a non-clinical sample (11), while we have argued that regret may play a special role in the psychopathology of depression based on a review of the evidence and theoretical considerations (25).

The present study seeks to examine individual differences in decision-making associated with two traits that are relevant to psychiatric disorders but are also variably present in the general population: hedonic capacity and trait anxiety. In particular, these traits are closely linked to major depressive disorder and anxiety disorders, which together carry a major global disability burden (26, 27). Much of the disability associated with these disorders likely stems from core deficits in appropriately assessing and balancing rewards and risks in routine situations. Developing a more accurate taxonomy of decision-making processing dysfunctions linked to hedonic capacity and trait anxiety could help to create more effective and specific interventions to improve functioning. In developing such a taxonomy, a key distinction concerns whether decision-making differences are rooted in differences in the subjective utility curve or are independent of subjective utility. Determining whether decision-making differences are related to differences in subjective utility or not will help to understand the mechanistic underpinnings of these differences and guide the development of interventions to improve decision-making.

Specifically, our objectives in this study were (a) to measure subjective utility via self-report, allowing us to determine the shape of the subjective utility curve at the individual level and relate this shape to individual risk preferences; (b) to relate hedonic capacity to subjective utility and to individual risk preferences, while determining whether any relationship between hedonic capacity and risk preferences was mediated by the shape of the subjective utility curve; (c) similarly, to relate trait anxiety to subjective utility and risk preferences; and (d) to measure the influence of counterfactual outcomes on subjective utility (i.e., to measure counterfactual sensitivity at the individual level) and to relate this to hedonic capacity and trait anxiety. In order to measure subjective utility, we designed an experimental decision-making task and asked subjects to rate their satisfaction after each outcome (on a visual analog scale). This same task allowed us to measure counterfactual influences on subjective utility as well as risk behaviors, based on subject choices between low- and high-risk options.

In line with our objectives, we hypothesized that (a) consistent with Daniel Bernoulli’s original proposal, the subjective utility curve would be concave, and that the concavity of the subjective utility curve would be associated with risk aversion across individuals; (b) hedonic capacity would be associated with higher subjective utility for gains and a less concave subjective utility function, and that this would result in more risk seeking behavior for individuals with greater hedonic capacity; (c) trait anxiety would be associated with risk aversion independently of any influence on the shape of the subjective utility curve; and (d) subjects with lower hedonic capacity and higher trait anxiety would exhibit greater regret-proneness as measured by self-report, as well as greater counterfactual sensitivity on the experimental task (i.e., counterfactual outcomes would exert a stronger influence on subjective utility).

## Materials and Methods

### Participants

78 college students (age range: 18–33; 21 males/57 females) participated in this study (approved by the Human Research Protections Program at University of California, San Diego). They were recruited from UCSD through an online system as part of lower division psychology classes during the summer and fall of 2013 and winter of 2014. They were contacted and scheduled for an experiment session. All participants signed informed consent and were compensated two course credits for completing the study.

### Measures

Prior to performing the experimental task, subjects completed self-report measures. These included the Snaith–Hamilton Pleasure Scale (SHAPS), a reliable and valid instrument to assess hedonic capacity (defined as capacity to take pleasure in typically rewarding experiences) (28). Higher SHAPS scores indicate higher hedonic capacity. Subjects also completed the Stait-Trait Anxiety Inventory, Trait scale (29) and a five-item measure of regret-proneness developed by Schwartz et al. (Regret Scale) (11).

### Decision-making Task

**Figure 1:**
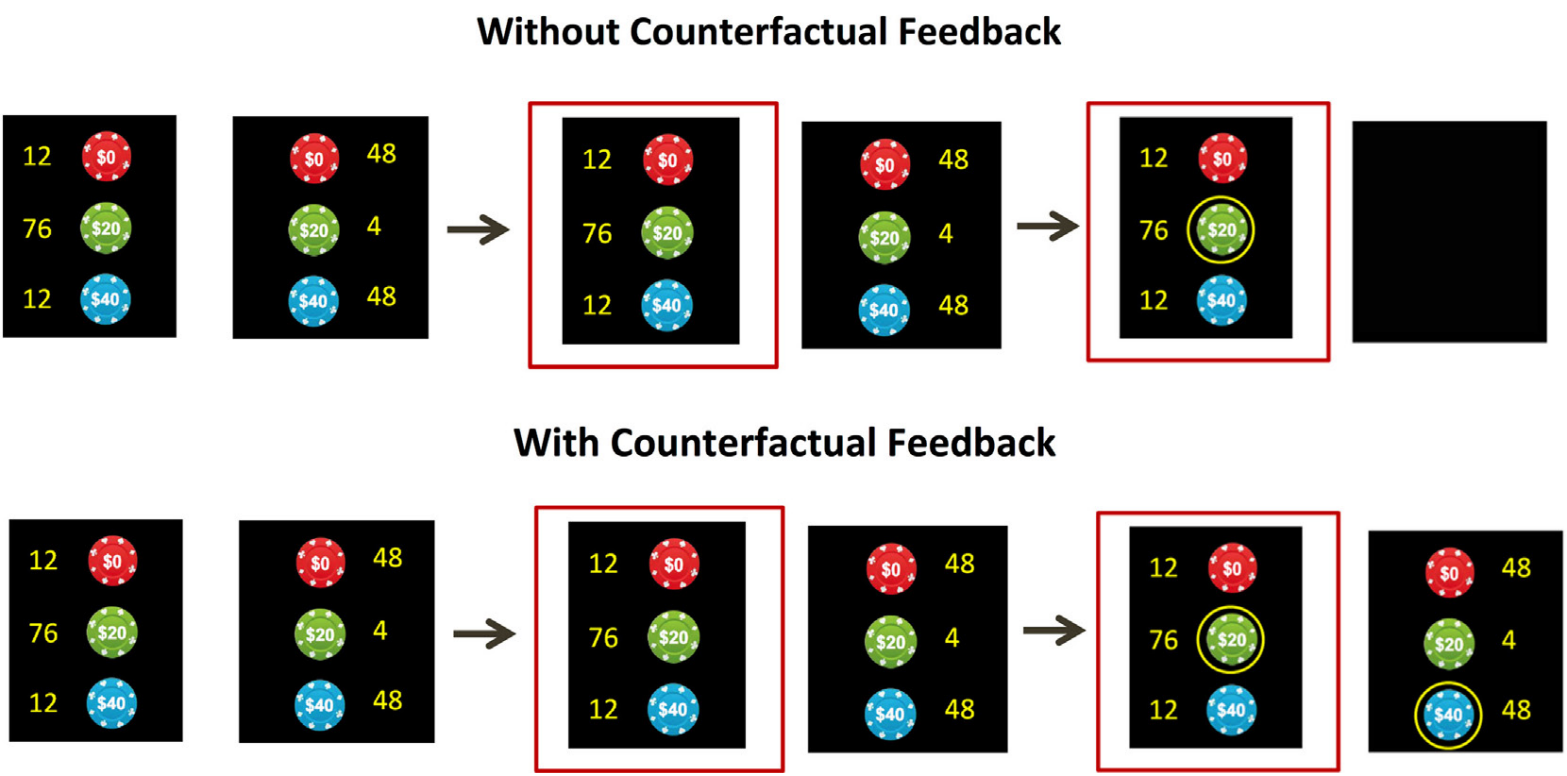
**The decision-making task**. On each trial, subjects were asked to imagine they were choosing between two random drawings of chips worth 0 points, 20 points, and 40 points. The yellow numbers indicate the number of each chip out of 100 (thereby presenting the probability of each outcome). Subjects chose between low-risk options (with a large number of 20-point chips) and high-risk options (with large numbers of 0-point and 40-point chips). Subjects were then shown the outcome of the choice. In some blocks, they were also shown the counterfactual outcome (the amount they would have received if they had made the opposite choice), while in other blocks this was not shown. After each trial, subjects indicated their satisfaction level on a visual analog scale.

Subjects completed a decision-making task consisting of 8 blocks with 24 trials per block. On each trial, subjects chose one of two gambles. They were asked to imagine they were choosing between two different random drawings with different numbers of chips worth 0, 20, or 40 points (subjects played for virtual points rather than real money). For each of the two options, subjects were shown the number of each type of chip (out of a total of 100). In this way, subjects were shown the value and probability of each possible outcome. Half of gambles were high risk (mathematical variance 384; larger numbers of 0-point and 40-point chips) and half were low risk (mathematical variance 96; larger number of 20-point chips). On average, both high-risk and low-risk gambles had the same expected values. After making a selection, subjects were shown the outcome of their choice (either 0, 20, or 40 points, which was determined by a random number generator in accordance with the stated probabilities). Half of blocks were “Counterfactual Feedback” blocks, in which subjects were shown what they would have received if they had made the opposite choice. The other half were “No Counterfactual Feedback” blocks, in which subjects were not shown the outcome of the opposite choice. The order of “Counterfactual Feedback” and “No Counterfactual Feedback” blocks was counterbalanced between subjects. After receiving feedback, subjects were asked to indicate their level of satisfaction with their choice on a visual analog scale. Refer to Figure 1 for a visual representation of the task.

### Data Analytic Approach

Subjects whose satisfaction ratings were not dependent on outcome were excluded from analysis.

For each subject, we calculated mean subjective utility of 0, 20, and 40 points. Subjective utility was determined by satisfaction ratings on the visual analog scale after each outcome. To test the hypotheses that hedonic capacity, but not trait anxiety, would be associated with higher subjective utilities for gains, we constructed linear regression models in R with these subjective utilities as dependent variables and either SHAPS or STAI-T as predictors, along with gender as a covariate. We performed Bonferroni adjustments to correct for multiple comparisons.

The subjective utility curve for each subject was constructed by plotting points (0, 20, and 40) on the *x*-axis and mean subjective utility on the *y*-axis. The subjective utility curve would be linear if the mean subjective utility of 20 falls on a straight line between the mean subjective utility of 0 and 40; if the mean subjective utility falls above this line, the subjective utility curve is concave. As a measure of the concavity of the subjective utility curve for each subject, we calculated the relative subjective utility of 20 points using the following formula: 40 × (mean utility of 20 − mean utility of 0)/(mean utility of 40 − mean utility of 0). The ratio of the difference in utility between 20 points and 0 points and the difference in utility between 40 points and 0 points measures the marginal utility of the first 20 points relative to the marginal utility of the entire 40 points. This will be 50% if the subjective utility curve is linear and greater than 50% if the subjective utility curve is concave. This value is then multiplied by 40 so that the result can be interpreted on the same scale as the original point scale. If the subjective utility curve is linear, then relative subjective utility of 20 points will be 20; if it is concave, then relative subjective utility of 20 will be greater than 20. To test the hypothesis that hedonic capacity, but not trait anxiety, would be associated with a less concave subjective utility curve, we constructed linear regression models in R with relative utility of 20 as a dependent variable and either SHAPS or STAI-T as predictors, with gender as a covariate. We performed Bonferroni adjustments to correct for multiple comparisons.

To assess risk preferences for each subject, we calculated a measure of risk seeking as follows: on trials in which subjects chose between low- and high-risk gambles, we calculated the percentage of trials on which they chose the high-risk gamble. Subjects who are low on this measure of risk seeking could be termed risk averse. To test the hypotheses that hedonic capacity would be associated with risk seeking, while both trait anxiety and the concavity of the subjective utility curve would be associated with risk aversion (i.e., would be negatively associated with risk seeking), we constructed linear regression models in R with risk seeking as a dependent variable and either relative utility of 20, SHAPS score, or STAI-T score as predictors and gender as a covariate. Additionally, we tested the hypotheses that trait anxiety, but not hedonic capacity, would be independently associated with risk preferences after controlling for the shape of the subjective utility curve. In order to test these independent effects of trait anxiety and hedonic capacity on risk preferences after controlling for the shape of the subjective utility curve, we constructed linear regression models in R with risk seeking as the dependent variable and relative utility of 20 along with SHAPS score or STAI-T score as predictors and with gender as a covariate. We performed Bonferroni adjustments to correct for multiple comparisons.

In order to test our hypotheses that hedonic capacity would be negatively associated with regret-proneness and that trait anxiety would be positively associated with regret-proneness, we constructed linear regression models in R with Regret Scale as a dependent variable and either STAI-T or SHAPS as predictors, with gender as a covariate. We performed Bonferroni adjustments to correct for multiple comparisons.

As a measure of counterfactual sensitivity, for each subject we calculated the difference in mean subjective utility of 20 points when the counterfactual outcome was 0 points compared to when the counterfactual outcome was 40 points. To further test our hypotheses that hedonic capacity would be negatively associated with counterfactual sensitivity and that trait anxiety would be positively associated with counterfactual sensitivity, and to determine the relationship between counterfactual sensitivity as measured by our experimental task with regret-proneness measured by self-report, we performed Pearson correlation tests between counterfactual sensitivity and the Regret Scale, STAI-T, and SHAPS, along with Bonferroni adjustments to correct for multiple comparisons.

## Results

Data from seven subjects were excluded because rated satisfaction was not dependent on outcomes.

Refer to Table 1 for associations between STAI-T and SHAPS and subjective utility of 0, 20, and 40 points. SHAPS was not associated with subjective utility of 0 but was positively associated with subjective utility of 20 [β = 0.33, *t*_(68)_ = 2.9, *p* = 0.006] and subjective utility of 40 [β = 0.27, *t*_(68)_ = 2.3, *p* = 0.03]. STAI-T was negatively associated with subjective utility of 0 [β = −0.28, *t*_(68)_ = −2.4, *p* = 0.02], subjective utility of 20 [β = −0.27, *t*_(68)_ = −2.3, *p* = 0.02], and subjective utility of 40 [β = −0.25, *t*_(68)_ = −2.1, *p* = 0.04]. However, only the association between SHAPS and subjective utility of 20 survived correction for multiple comparisons.

**Table 1:** **Associations between Snaith–Hamilton Pleasure Scale (SHAPS) and State-Trait Anxiety Inventory, Trait scale (STAI-T) and mean subjective utility of winning 0, 20, or 40 points, as measured by satisfaction ratings on a visual analog scale**.

	Subjective utility of 0	Subjective utility of 20	Subjective utility of 40
SHAPS	Not associated	β = 0.33, *t*_(68)_ = 2.9, *p* = 0.006	β = 0.27, *t*_(68)_ = 2.3, *p* = 0.03
STAI-T	β = −0.28, *t*_(68)_ = −2.4, *p* = 0.02	β = −0.27, *t*_(68)_ = −2.3, *p* = 0.02	β = −0.25, *t*_(68)_ = −2.1, *p* = 0.04

*Only the association between SHAPS and subjective utility of 20 survived correction for multiple comparisons*.

**Figure 2:**
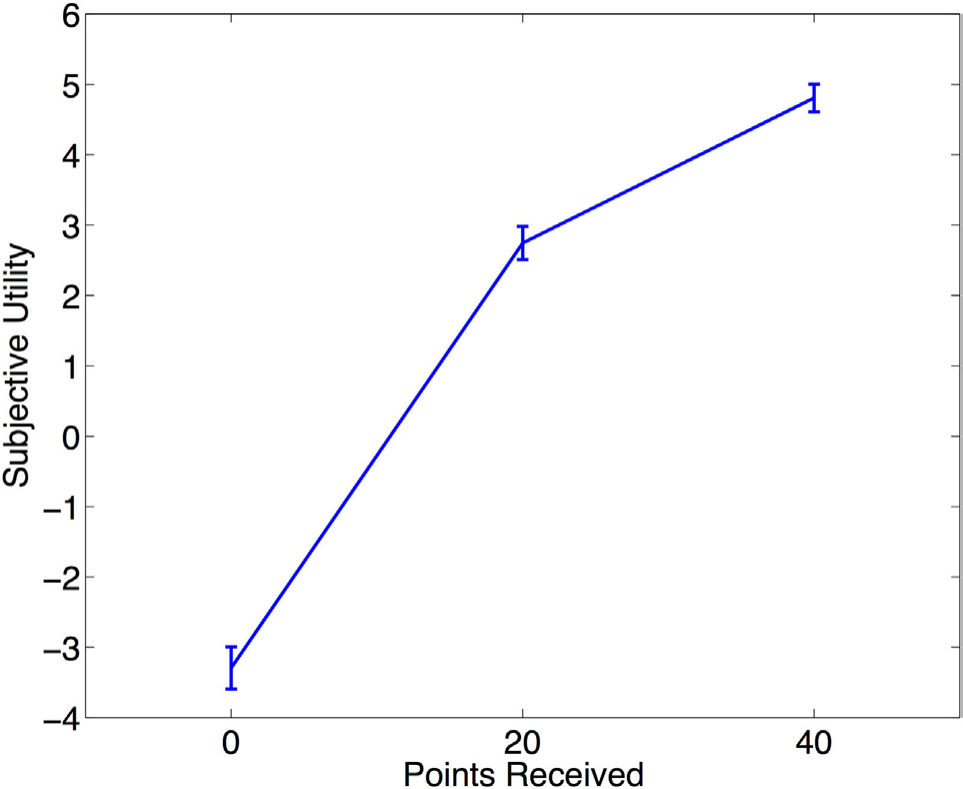
**The subjective utility curve**. Mean subjective utility of receiving 0, 20, and 40 points based on satisfaction ratings on a visual analog scale. The shape of the subjective utility curve is concave. Error bars represent standard error of the mean.

Across subjects, relative subjective utility of 20 was significantly higher than 20 (*p* = 2 × 10^−16^). This indicates that the subjective utility curve was concave. Neither STAI-T nor SHAPS were associated with the relative subjective utility of 20. Refer to Figure 2 for a plot of the subjective utility curve.

**Figure 3:**
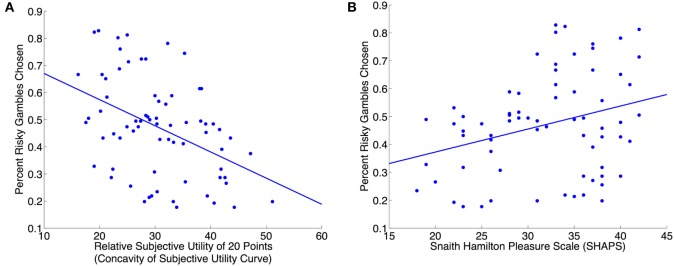
**Concavity of the subjective utility curve is associated with risk aversion while hedonic capacity is associated with risk seeking. (A)** Relative subjective utility of 20, a measure of the concavity of the subjective utility curve, is negatively associated with risk seeking [β = −0.37, *t*_(68)_ = −3.5, *p* = 0.001]. Relative subjective utility of 20 points is calculated as: 40 × (mean subjective utility of 20 − mean subjective utility of 0)/(mean subjective utility of 40 − mean subjective utility of 0). **(B)** Hedonic capacity is positively associated with risk seeking [β = 0.29, *t*_(68)_ = 2.6, *p* = 0.01]. Hedonic capacity was measured using the self-report Snaith–Hamilton Pleasure Scale (SHAPS). The association between hedonic capacity and risk seeking was independent of the shape of the subjective utility curve; a linear regression with risk seeking as the dependent variable and relative subjective utility of 20 and SHAPS as predictors (with gender as a covariate) found a significant main effect of SHAPS [β = 0.34, *t*_(67)_ = 3.4, *p* = 0.001].

The relative subjective utility of 20 was negatively associated with risk seeking [β = −0.37, *t*_(68)_ = −3.5, *p* = 0.001, Figure 3A]. SHAPS score was positively associated with risk seeking [β = 0.29, *t*_(68)_ = 2.6, *p* = 0.01, Figure 3B]. STAI-T was not associated with risk seeking.

A linear regression model with risk seeking as the dependent variable and the relative subjective utility of 20 and SHAPS as predictors (with gender as a covariate) determined that the relative subjective utility of 20 was negatively associated with risk seeking [β = −0.41, *t*_(67)_ = −4.1, *p* = 0.0001] and SHAPS was positively associated with risk seeking [β = 0.34, *t*_(67)_ = 3.4, *p* = 0.001]. A linear regression model with risk seeking as the dependent variable and the relative subjective utility of 20 and STAI-T as predictors (with gender as a covariate) showed that STAI-T was not associated with risk seeking after controlling for the relative subjective utility of 20.

STAI-T was positively associated with the Regret Scale [β = 0.51, *t*_(68)_ = 5.0, *p* = 4 × 10^−6^]. SHAPS was not associated with the Regret Scale.

**Figure 4:**
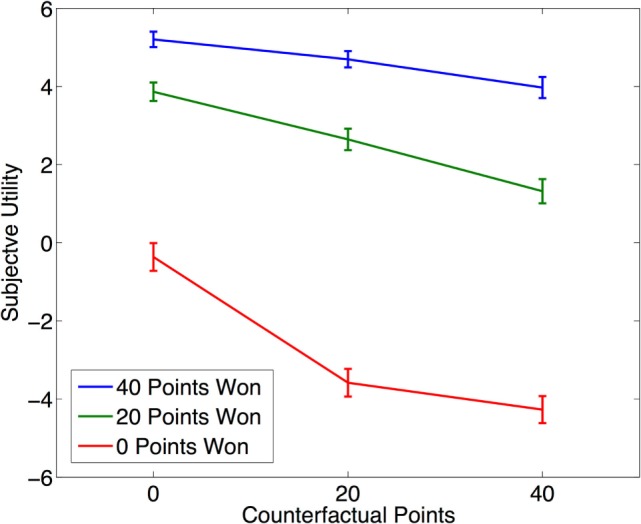
**Influence of counterfactual outcomes on subjective utility**. Subjective utility was based on satisfaction ratings after each trial on a visual analog scale. Subjective utility of any outcome (0, 20, or 40 points) was influenced by counterfactual outcomes. Higher counterfactual outcomes (e.g., 40 points) were associated with lower subjective utility of the actual outcome. Error bars represent standard error of the mean.

Subjective utility of 20 points with counterfactual outcome of 0 points was significantly greater than with counterfactual outcome of 40 points (*p* = 5 × 10^−15^), showing that counterfactual outcomes had a significant influence on subjective utility. However, none of the self-report measures (Regret Scale, STAI-T, or SHAPS) were associated with counterfactual sensitivity. Refer to Figure 4 for a plot showing the influence of counterfactual outcomes on subjective utility.

## Discussion

This study aimed to delineate the influence of hedonic capacity and trait anxiety on decision-making and whether or not these influences occurred via an effect on the subjective utility curve. Separating decision-making effects into effects on subjective utility vs. other factors could help to understand the neural basis of these effects and to develop interventions to improve decision-making in psychiatric disorders and in normal individuals. We measured subjective utility directly by asking subjects to rate their satisfaction level after each outcome in a decision-making task. The results demonstrate that this measure of subjective utility was relevant for understanding decision-making: as expected, the subjective utility curve was concave, and the concavity of the subjective utility curve was associated with risk aversion. These observations are consistent with Daniel Bernoulli’s proposal that concavity of the subjective utility curve explains risk aversion in decision-making (5).

Consistent with our expectations, hedonic capacity was associated with increased risk seeking behavior on the decision-making task. Similarly, depression has previously been linked to risk aversion on the Iowa Gambling Task (30). These findings are consistent with the idea that risk taking depends on the ability to anticipate and enjoy large, rewarding gains. Hedonic capacity did increase the subjective utility of winning 20 points. However, unexpectedly, hedonic capacity was not associated with a more linear subjective utility curve, and the association between hedonic capacity and risk seeking was not dependent on the shape of the subjective utility curve. There are several possible explanations for a relationship between hedonic capacity and risk seeking that is independent of the shape of the subjective utility curve. A better understanding of the mechanisms underlying this relationship would be relevant to the pathophysiology of depression, because depressed individuals are known to have low hedonic capacity. First, it is possible that the subjective utility curve during anticipation of outcomes while making decisions differs from the subjective utility curve during consummation of the outcome (which we measured on this task). Anticipation and consummation of reward are dissociable processes and may rely on somewhat different neural circuitry (31). Future research could potentially map separate utility curves during anticipation and receipt using functional neuroimaging of reward-sensitive brain areas and determine their specific relationships to risk preferences in the context of hedonic capacity. A second possibility is that subjects differed in their weighting of probabilities. Individuals with both depression and anxiety may differ in respect to probability weighting in addition to subjective utilities (32). Another possibility is that these subjects exhibit a different attitude toward risk *per se* that is not mediated by either subjective utility or probability weighting. Finally, it is possible that the relationship between hedonic capacity and risk seeking is in fact mediated by the shape of the subjective utility function, but in a manner that we were unable to measure. Future research with decision-making tasks including a broader range of outcomes, leading to a more fine-grained view of the subjective utility curve, may help test this possibility.

Surprisingly, trait anxiety was not associated with risk aversion on this task. Trait anxiety has previously been linked to risk aversion on the balloon analog risk task (BART), where subjects receive points for inflating a virtual balloon as much as possible without exceeding an unknown “explosion” threshold (in which case they lose all points) (33). An important difference between the BART and the task in the present study is that with the BART there is a possibility of losses (losing gains that have already been made). Therefore, unlike the task in the present study, it is not a specific measure of risk aversion for gains. Another study measured the association between depression and anxiety with risk aversion on a task in which subjects faced hypothetical decisions that could have positive or negative outcomes (gains and losses). Both anxiety and depression were associated with risk aversion, but the relationship between depression and risk aversion was mediated by anxiety. Again, the study did not specifically test risk aversion for gains (34). Anxiety and stress may also affect risk behavior via influence on the subjective framing of decisions (21–23).

The Regret Scale, a self-reported measure of regret-proneness, was associated with trait anxiety but not hedonic capacity. A previous study with a non-clinical sample reported that the Regret Scale was correlated with the Beck Depression Inventory, a measure of depression severity rather than anhedonia specifically (11). However, contrary to our expectations, counterfactual sensitivity on our decision-making task was unrelated to the Regret Scale, trait anxiety, or hedonic capacity. In general, subjects were sensitive to counterfactual effects as expected (i.e., better counterfactual outcomes led to lower subjective utility of an actual outcome). The lack of association between counterfactual sensitivity on the task and the clinically relevant self-report scales suggests that counterfactual sensitivity may in fact be a multidimensional construct. The influence of counterfactual outcomes after small decisions may be unrelated to regret over larger decisions. This regret over larger, more significant decisions, as captured by the Regret Scale, appears to be more clinically relevant. Rather than immediate counterfactual influences on subjective utility as measured by our task, the relationship between counterfactual outcomes and psychopathology may be mediated through other pathways. For example, individuals with depression may be more likely to ruminate over time about a regretted outcome or to experience self-esteem damage as a result of significant regret (25). These processes may not have been adequately measured by our task.

Our decision-making task only included gains as outcomes rather than losses. According to Prospect Theory, a classic behavioral economic account, people are typically risk seeking for losses (unlike for gains) (35). This is explained by a convex subjective utility curve for losses, contrasting with the concave subjective utility curve for gains. Future research can use methods similar to those used here in order to measure the subjective utility curve for losses and relate it to risk behavior. Additionally, future results can provide more detailed information about the subjective utility curve by using a larger number of different point outcomes (both for losses and for gains).

Future research using functional neuroimaging can examine the neural underpinnings of the observed effects. Subjective utility signals have been localized to brain regions involved in reward processing (36). For example, the dorsal striatum encodes the subjective utility of an outcome rather than simply the objective reward magnitude (37). Evidence indicates that probability estimates are separately computed in the medial prefrontal cortex (MPFC) (38). Anhedonia has been associated with decreased activation of the MPFC and specifically ventromedial prefrontal cortex (VMPFC) in response to positive stimuli (16, 39). Additionally, evidence indicates that aversion to loss is exaggerated in depression and that this difference is associated with altered activation of right dorsal striatum, right anterior insula, and ventral tegmental area (40). Anxious individuals are likely to exaggerate the probability of a negative outcome (3, 20), an effect which may be related to decreased activation in VMPFC and ventral striatum and increased activation of the insula when making risky decisions (41). Trait anxiety may be associated with greater influence of emotions on decision-making, mediated by increased amygdala activity (22). Mapping response in reward-sensitive brain regions to different outcomes in individuals with different levels of hedonic capacity, and relating this to individual risk preferences, may further elucidate neural dysfunction and its connection to behavioral disturbances. Separate maps can be constructed for anticipation of outcome vs. consummation of reward, and these value functions can be separately tested for association with risk preferences. Activity in reward-sensitive brain regions, including MPFC, is also sensitive to counterfactual effects (i.e., superior counterfactual outcomes result in lower activation in reward-sensitive regions) (42). Additionally, orbitofrontal cortex (OFC) is activated in response to counterfactual outcomes, while patients with OFC lesions show a lack of affective influence of counterfactual outcomes (43, 44). Investigating differences in brain activations in response to counterfactual outcomes of different magnitudes in individuals with different levels of hedonic capacity and trait anxiety may clarify neural processing differences in these individuals.

The current results show that subjective utility can be usefully measured through self-reported satisfaction and that hedonic capacity is associated with risk seeking independently of the shape of the subjective utility curve (despite classical economic accounts attributing risk preferences to the shape of the subjective utility curve). This finding could be accounted for by an effect on reward anticipation as opposed to consummation, by an alteration in probability judgments, or by an effect on “pure” risk preferences. While further research will be needed to more fully examine the mechanism of the relationship between hedonic capacity and risk seeking, the results have implications for understanding the biological basis of individual variability in decision-making. Unexpectedly, trait anxiety was not associated with risk aversion, suggesting that hedonic capacity may play a more important role in risk behaviors. Additionally, the influence of counterfactual outcomes on subjective utility did not vary based on hedonic capacity or trait anxiety. Further investigations into the underlying mechanisms of individual differences in decision-making, based on theoretical frameworks from classical and behavioral economics, promise to help develop interventions to improve decision-making in psychiatric conditions and in the general population.

## Ethics Statement

This study was approved by the University of California, San Diego Human Research Protections Program. Subjects provided written informed consent.

## Author Contributions

JH and MP together conceived of, designed, carried out, and analyzed the study and wrote the paper.

## Conflict of Interest Statement

The authors declare that the research was conducted in the absence of any commercial or financial relationships that could be construed as a potential conflict of interest.
